# Microbiome Stability in Wild and Rehabilitated Insectivorous Bats Revealed by Shotgun Metagenomics

**DOI:** 10.3390/microorganisms14071403

**Published:** 2026-06-25

**Authors:** Dongsheng Luo, Alise J. Ponsero, Kate Wright, David J. Baker, Andrea Telatin, Colin Townsley, Efstathios S. Giotis

**Affiliations:** 1 School of Life Sciences, University of Essex, Colchester CO4 3SQ, UK; dl25703@essex.ac.uk; 2 Quadram Institute Bioscience, Norwich Research Park, Norwich NR4 7UQ, UK; 3 West Yorkshire Bat Group, Bradford BD9 4RX, UK; 4 Department of Infectious Diseases, School of Medicine, Imperial College London, London SW7 2AZ, UK

**Keywords:** bat microbiome, metagenomics, microbial ecology, rehabilitation, *Myotis daubentonii*, *Pipistrellus pipistrellus*, gut microbiota

## Abstract

Wildlife rehabilitation can alter host-associated microbial communities, yet the effects of temporary managed care on the gut microbiome of insectivorous bats remain poorly understood. We used shotgun metagenomic sequencing to investigate gut microbiome composition in wild and rehabilitated bats from Yorkshire, United Kingdom. A total of 25 faecal metagenomes were analysed from four bat species (*Myotis daubentonii*, *Pipistrellus pipistrellus*, *Nyctalus noctula*, and *Nyctalus leisleri*), including wild baseline individuals and bats undergoing temporary managed care for 1–49 days. Microbial community structure clustered primarily according to host species and roost location, with no significant separation associated with rehabilitation status. Among bats in managed care, bacterial alpha diversity did not differ significantly with time in care (H = 2.30, *p* = 0.32). Archaeal communities displayed markedly lower interindividual variation than bacterial communities (coefficient of variation: 12.2% vs. 41.8%), indicating a highly conserved archaeal microbiome across hosts. Rehabilitated bats exhibited modest compositional shifts in bacterial communities, including increased relative abundances of *Yersiniaceae* and *Lactobacillaceae* and reduced abundances of environmentally associated taxa such as *Pseudomonadaceae* and *Erwiniaceae*. These changes may reflect controlled dietary provision and reduced environmental exposure during care. Overall, no marked rehabilitation-associated differences in gut microbiome diversity or community structure were detected under the current sampling design. These findings are consistent with microbiome stability during temporary managed care, although longitudinal studies are required to confirm microbiome dynamics within individual bats. Nonetheless, this study provides an initial baseline for future microbiome-informed wildlife rehabilitation studies.

## 1. Background

Bats are ecologically vital and legally protected species in the United Kingdom, contributing to insect population control, pollination, and broader ecosystem balance [1]. However, many species are increasingly threatened by habitat loss, food scarcity, and anthropogenic pressures that result in injury, illness, or displacement [2,3]. The gut microbiome is central to animal health, mediating nutrient absorption, immune regulation, and resistance to pathogens [4]. In mammals, captivity and dietary change can substantially alter microbial diversity and composition, often reducing ecological complexity and enriching opportunistic taxa [5,6,7,8].

Despite the importance of rehabilitation in conservation programmes, the effects of short-term rehabilitation and controlled diets on the gut microbiome of insectivorous bats remain unexplored [9,10,11,12]. This gap is particularly relevant given the limited understanding of the role of the bat gut microbiome in pathogen tolerance, and that certain bat species harbour a wide range of viruses, including coronaviruses, rhabdoviruses, paramyxoviruses, and influenza-like viruses, reflecting their long evolutionary associations with diverse viral families [13,14,15,16,17]. Rehabilitation feeding typically relies on mealworms and other commercially reared insects, providing a simplified diet that may alter microbial composition relative to that of wild prey [18].

In the UK, no previous studies have examined gut microbiome responses of insectivorous bats during rehabilitation. This represents a critical gap, as temporary captivity is common in bat conservation practice. Understanding whether short-term rehabilitation alters microbial composition is essential for ensuring welfare standards, maintaining ecological function, and supporting post-release adaptation [12]. In this study, we used shotgun metagenomic sequencing to investigate the gut microbiome of four insectivorous bat species, *Myotis daubentonii*, *Pipistrellus pipistrellus*, *Nyctalus noctula*, and *N. leisleri*, from wild roosts and rehabilitation facilities in Yorkshire, UK. We compared microbial diversity and composition between wild bats and bats sampled during temporary managed care and assessed whether microbiome patterns varied with time in care (1–49 days).

## 2. Methods

### 2.1. Study Design and Sampling

Thirty-two faecal DNA extracts were initially collected from insectivorous bats across Yorkshire, UK, including individuals sampled from wild roosts and rehabilitation facilities coordinated by the West Yorkshire Bat Group (Supplementary Table S1). Sampling was non-invasive, with fresh faecal pellets collected from clean surfaces beneath roosts or within holding enclosures during rehabilitation. Following quality filtering (minimum 1 million post-QC reads), seven bat samples were excluded for insufficient read depth, leaving 25 samples for analysis: 14 from wild baseline bats and 11 from bats sampled during managed care (Supplementary Tables S2 and S3). Rehabilitation duration was determined from admission and sampling records provided by bat carers registered with the Bat Conservation Trust. Bats in managed care were sampled between 1 and 49 days after admission. Most samples (C01–C03 and C07–C11) were collected within 7 days of care, whereas three samples (C04–C06) were collected after extended care of 42–49 days. Samples labelled W represent wild baseline bats sampled directly from roosts. Species-level identity was resolved for 19 of the 25 samples from mitochondrial contigs and reference-genome mapping (see methods below). Using both approaches, four bat species were detected: *Myotis daubentonii*, *Pipistrellus pipistrellus*, *Nyctalus noctula* and *Nyctalus leisleri*.

### 2.2. DNA Sequencing and Processing

Metagenomic libraries were prepared using standard Illumina protocols and sequenced on a NovaSeq X Plus platform (25B flow cell) at Quadram Institute to generate paired-end 150 bp reads. A library preparation blank (no-template control) was included; it yielded no quantifiable library and was therefore not sequenced. No field blanks were collected, as samples were obtained non-invasively as fresh faecal pellets. Raw reads were quality-trimmed with TrimGalore (v0.6.10), to remove low-quality bases and adapter sequences. Human-associated contamination was filtered using Hostile (v2.0.0) with the human-t2t-hla reference [19]. To ensure sufficient sequencing depth for reliable profiling analysis, a threshold of 1 million reads post-QC was applied. Sequencing data have been deposited in the European Nucleotide Archive under accession number PRJEB103765.

### 2.3. Metagenomic Analysis

Host identification: Bat host identity was determined by combining two complementary signals: a genus-level signal from read mapping to reference genomes, and a species-level identification from mitochondrial sequence matches. Quality-controlled reads were assembled with MEGAHIT v1.2.9 and contigs classified as organellar or non-organellar using Tiara v1.0.3. Organellar contigs were queried against the NCBI nucleotide database (nt v5) using BLASTn v2.10 (alignment length ≥ 500 bp, e-value ≤ 1 × 10^−10^, percent identity ≥ 95%); the top-scoring mitochondrial match provided species-level identification.

To complement this, quality-controlled reads were also mapped against complete bat reference genomes to obtain a genus-level signal. A panel of UK-resident or commonly recorded bat species was compiled from the Bat Conservation Trust (https://www.bats.org.uk/, accessed on 10 October 2025), and reference genomes were retrieved from NCBI for as many species as possible, substituting the closest available relative where the target species genome was unavailable. The panel comprised *Myotis daubentonii* (GCF_963259705.1), *Myotis brandtii* (GCF_000412655.1), *Myotis mystacinus* (GCA_964094495.3), *Myotis myotis* (GCF_014108235.1), *Pipistrellus pipistrellus* (GCA_965203405.2), *Pipistrellus pygmaeus* (GCA_949987585.2), *Pipistrellus nathusii* (GCA_963693515.1), *Nyctalus leisleri* (GCA_964264875.2), *Eptesicus fuscus* (GCF_027574615.1), *Plecotus auritus* (GCF_963455305.1), *Rhinolophus ferrumequinum* (GCF_004115265.2), *Rhinolophus hipposideros* (GCF_964194185.1), and *Cnephaeus nilssonii* (GCF_951640355.1); reference genomes were not available for *Barbastella barbastellus*, *Eptesicus serotinus*, *Myotis alcathoe*, *Myotis bechsteinii*, *Nyctalus noctula*, and *Plecotus austriacus*. Read mapping was performed with Bowtie2 v2.5.4. The proportion of reads mapping to each reference was calculated for all samples and displayed as a heatmap with hierarchical clustering (sample-wise z-score normalisation; Euclidean distance, complete linkage, for both samples and references) using pheatmap v1.0.12, and the resulting clusters were used to infer the most likely host genus.

A sample was assigned to species only when the mitochondrial match and the genus-level mapping signal were concordant. Where the two were discordant, or where only one signal was available (for example, mitochondrial recovery without a supporting mapping cluster, or a mapping signal without recoverable mitochondrial sequence), the sample was left unassigned. Species lacking a reference genome were identifiable at species level through this scheme provided their reads clustered with a congeneric reference at genus level and a mitochondrial match was recovered; for example, *Nyctalus noctula* (no reference genome available) was resolved by mitochondrial match while clustering with the *Nyctalus leisleri* reference at genus level.

### 2.4. Prokaryotic Profiling

Quality-controlled reads were taxonomically classified using Kraken2 v2.1.3 against the k2_core_nt_20250609 database (build dated 9 June 2025 available at https://benlangmead.github.io/aws-indexes/k2, accessed on 10 October 2025). Abundance re-estimation with Bracken was not applied: because a substantial fraction of the community in these samples is expected to be absent from reference databases, Bracken’s redistribution of reads to reference taxa would impose species-level assignments not supported by the data and distort the resulting profiles. Analyses were therefore based on direct Kraken2 assignments aggregated at the family level. All statistical analysis and visualizations were performed in R v4.3.3.

Beta-diversity analysis: Family-level prokaryotic profiles were generated by combining bacterial and archaeal read counts, retaining reads assigned at the family level. No prevalence or abundance filtering was applied. For ordination, a pseudocount of 0.5 was added to all family counts and a centred log-ratio (CLR) transformation was applied per sample (the log of each family count minus the mean log count across all families in that sample); principal component analysis was performed on the resulting matrix (prcomp, no scaling), representing an Aitchison-distance ordination. Differences in community structure were tested by permutational multivariate analysis of variance (PERMANOVA; adonis2, vegan v2.7.3, 999 permutations) on Aitchison distances. Because host species, sampling location, and rehabilitation status are partially confounded by the opportunistic sampling design, a full additive model could not be fitted. We instead used two reduced models testing rehabilitation status conditional on each major confounder in turn (status after host species, restricted to the confirmed-species samples, *n* = 19; and status after location, all samples, *n* = 25), with status entered last under sequential testing so that its effect was estimated conservatively. Homogeneity of multivariate dispersion was assessed for each grouping factor using betadisper followed by permutest (999 permutations).

Alpha diversity analysis: the Shannon index was calculated on prokaryotic counts at the family level using the microbiome v1.28.0 and phyloseq v1.50.0 packages. To assess the effect of time in care, in-care bats were grouped by care duration into newly admitted (<4 days, *n* = 4), short-term care (4–7 days, *n* = 4), and long-term care (30+ days, *n* = 3), and Shannon diversity was compared across these groups using Kruskal–Wallis tests (bacteria and archaea separately), with pairwise Wilcoxon rank-sum tests (Bonferroni-corrected) to be applied where the overall test was significant. Wild-roost samples were not included in this comparison, as care status is confounded with host health, which would prevent attribution of any difference to care per se.

### 2.5. Eukaryotic Profiling

Quality-controlled reads were assembled with MEGAHIT v1.2.9 [20], and contigs were classified using Tiara v1.0.3 [21]. Organellar contigs were annotated by BLASTn against the NCBI nt database (v5) using the same filtering criteria (alignment length ≥ 500 bp, e-value ≤ 1 × 10^−10^, percent identity ≥ 95%), retaining the top-scoring hit for taxonomic assignment. For the fungal and arthropod presence/absence analyses, a genus was scored as present in a sample when at least one organellar contig was assigned to it under the BLASTn criteria above; genera with no qualifying contig were scored as absent.

### 2.6. Viral Profiling

Viral sequences were identified from assembled contigs using geNomad v1.8.0 [22]. Viral sequence quality was assessed with CheckV v1.0.3 (database v1.5) [23], retaining sequences classified as medium, high, or complete, or >1 kb in length. A total of 28,371 sequences were clustered into vOTUs (95% identity over 85% of the longest sequence) using aniclust and anicalc (CheckV utilities), yielding 22,729 vOTUs. Quality-controlled reads were mapped to representative vOTU sequences using bwa-mem as implemented in coverM v0.7.0, with a minimum coverage of 75% and read identity of 90%, and abundances were normalised as TPM using coverM v0.7.0 [24].

## 3. Results

**Figure 1:**
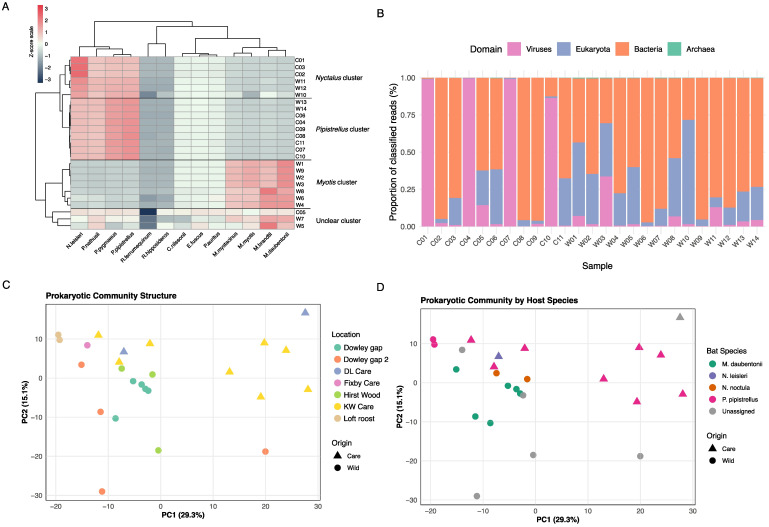
Overview of microbiome composition across wild and rehabilitated bats. (**A**) Heatmap of the proportion of quality-controlled reads from each faecal sample mapping to bat reference genomes. Values are z-score normalized by sample (rows) to highlight relative mapping preferences. Samples and reference genomes (columns) are hierarchically clustered using Euclidean distance and complete linkage. Samples marked as “unclear” show no distinct clustering pattern to any reference genome cluster, indicating insufficient host DNA content for genus assignment. (**B**) Domain-level composition of classified reads showing the relative proportions of Bacteria, Eukaryota, Viruses, and Archaea. (**C**) Principal component analysis (PCA) of prokaryotic community composition coloured by sampling location and shaped by origin (wild vs. care). (**D**) PCA coloured by host species and shaped by origin (wild vs. care). PCA was performed on centered log-ratio (CLR) transformed abundance data representing Aitchison distance.

After quality control and read-depth filtering (>1 × 10^6^ reads), 25 faecal metagenomes were retained for analysis, comprising 14 wild bat samples from natural roosts and 11 bat samples taken during rehabilitation. Host species identity was determined through mitochondrial genome assembly and reference mapping (Figure 1A, Supplementary Table S2). Species-level identification was achieved for 19 samples: 6 *Myotis daubentonii*, 10 *Pipistrellus pipistrellus*, 1 *Nyctalus leisleri*, and 2 *Nyctalus noctula*. An additional six samples could be provisionally assigned by reference-genome mapping but lacked sufficient mitochondrial sequence data for independent confirmation (Supplementary Table S2).

**Figure 2:**
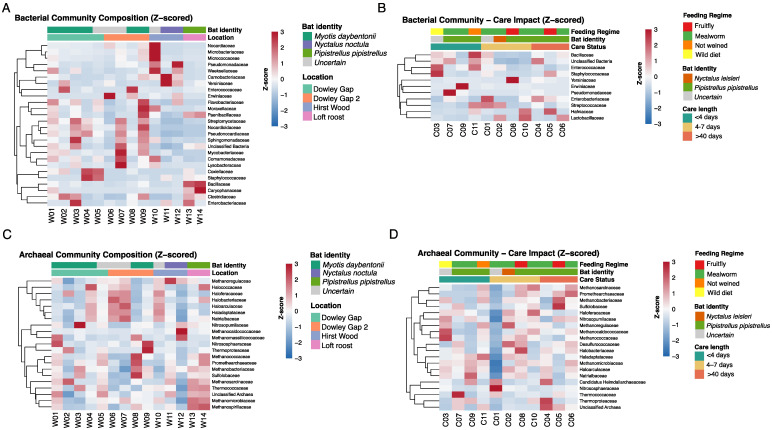
Bacterial and archaeal community composition in wild and managed-care bats. Z-scored relative abundances of dominant prokaryotic families (detected in >1% in ≥3 samples) across wild samples ((**A**) Bacteria; (**C**) Archaea) or bats in care ((**B**) Bacteria; (**D**) Archaea), annotated by host species and sampling location and feeding.

Prokaryotic sequences dominated the classified fraction of reads (median = 74.7%), followed by eukaryotic (17.3%) and viral sequences (1.6%) (Figure 1B, Supplementary Figure S1). Principal component analysis (PCA) of centred log-ratio-transformed family-level abundances revealed clear clustering by host species and sampling location, but no clear distinction between wild and in-care bats (Figure 1C,D). These factors were confounded in the study design, as species composition differed across sampling locations (e.g., all *M. daubentonii* originated from a specific location: Dowley Gap arches, Supplementary Table S1). Consistent with this, PERMANOVA on Aitchison distances showed that rehabilitation status did not explain a significant proportion of community variation, whether tested beyond host species (R^2^ = 0.08, F = 1.72, *p* = 0.078; confirmed-species samples, *n* = 19) or beyond sampling location (R^2^ = 0.03, F = 0.73, *p* = 0.712; *n* = 25), whereas host species (R^2^ = 0.23, *p* = 0.044) and sampling location (R^2^ = 0.38, *p* = 0.003) were each significantly associated with community structure. Multivariate dispersion was homogeneous between wild and in-care bats (permutest *p* = 0.41) but was heterogeneous among host species and among locations (*p* = 0.001 and *p* = 0.004, respectively), so these confounder effects reflect differences in both community centroid and within-group spread. As host species, location, and rehabilitation status are partially confounded in this dataset, these factors cannot be fully disentangled with this sampling design. Family-level community composition of wild-roost bats, grouped by host species and sampling location, is shown in Figure 2A (bacteria) and Figure 2C (archaea).

Among in-care bats grouped by time in care, bacterial alpha diversity did not differ significantly across care-duration groups (newly admitted, short-term, and long-term care; Kruskal–Wallis H = 2.30, *p* = 0.32), and neither did archaeal diversity (H = 0.66, *p* = 0.72; Supplementary Figure S1C). As the overall tests were non-significant, no pairwise comparisons were performed. However, among bats in care, descriptive compositional shifts were observed at the family level between newly admitted bats (<4 days) and those in short-term care (4–7 days) (Figure 2B,D). Mean relative abundance of Lactobacillaceae rose from 4.5% to 31.9%, and Yersiniaceae from 0.85% to 21.0%, whereas families commonly associated with environmental exposure declined: Erwiniaceae from 19.3% to 1.2%, and Pseudomonadaceae from 9.3% to 0.4% (Figure 2B). The enrichment of *Lactobacillaceae* may reflect acquisition of bacteria associated with the mealworm (*Tenebrio molitor*) gut microbiome, or the effects of milk powder supplementation. Given the limited sample size of the long-term rehabilitation group and the compositional nature of relative abundance data, these observations should be interpreted as trends rather than statistically supported differences.

In contrast to the modest compositional shifts observed in bacterial communities, archaeal communities were highly uniform across all samples, dominated by *Methanobacteriaceae* and *Methanomassiliicoccaceae*. Archaeal family-level profiles showed minimal inter-individual variation (12.2% coefficient of variation) compared to bacterial communities (41.8% coefficient of variation), and no clear separation between wild bats and bats in rehabilitation (Figure 2C,D). Archaeal diversity did not differ significantly according to the rehabilitation period length (Kruskal–Wallis H = 0.66, *p* = 0.72, Supplementary Figure S1C).

Fungal DNA was detected sporadically in 13 of 25 samples (52%), representing 23 genera dominated by *Penicillium*, *Mucor*, and *Debaryomyces*. Fungal reads were more frequent in wild roost samples (60%) than in rehabilitation facilities (38%), consistent with environmental or dietary origins rather than established gut colonisation. Parasitic DNA was identified in two wild bats: *Plagiorchis vespertilionis* and *Eimeria jerfinica*, both recognised bat parasites, and *Gordius albopunctatus*, a nematomorph likely derived from infected arthropod prey.

**Figure 3:**
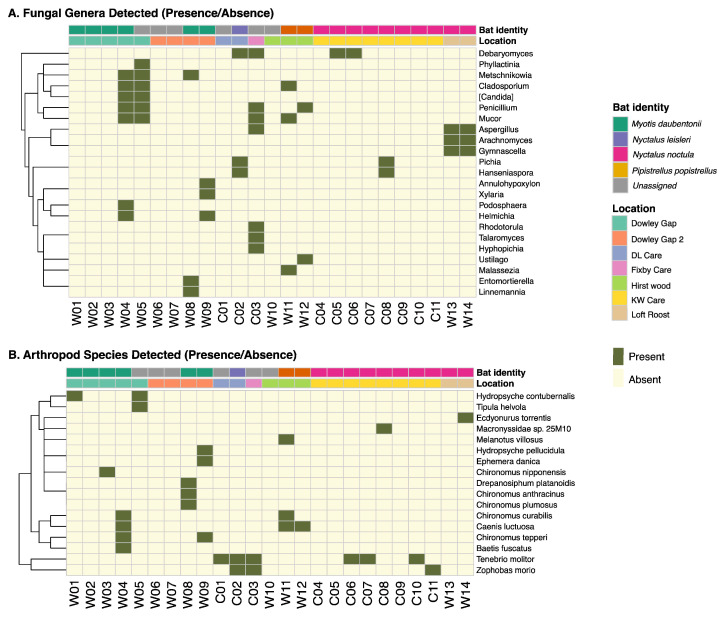
Fungal and arthropod sequences detected in wild and rehabilitated bats. Heatmap showing the distribution of (**A**) fungal genera and (**B**) arthropod genera across bat faecal samples based on organellar BLAST analysis. Each cell represents the presence (green) or absence (cream) of a fungal/arthropod genus in a given sample. Samples (columns) are ordered by collection location (top annotation bar) with bat host species indicated (second bar). Genera (rows) are hierarchically clustered using Euclidean distance to group taxa with similar distribution patterns.

Arthropod mitochondrial sequences revealed clear differences in prey composition between wild and captive bats (Figure 3B). Wild individuals consumed a variety of aquatic and semi-aquatic insects, including mayflies (*Caenis luctuosa*, *Ephemera danica*), caddisflies (*Hydropsyche contubernalis*), and crane flies (*Tipula helvola*), consistent with foraging over water. In contrast, bats in rehabilitation fed almost exclusively on commercial feeder insects (*Tenebrio molitor*), validating dietary records provided by carers.

The assembled viral catalogue comprised 22,729 viral operational taxonomic units (vOTUs), dominated by bacteriophages of the realm *Duplodnaviria* (≈90%), followed by *Varidnaviria*, *Monodnaviria*, and *Riboviria* (Supplementary Figure S2). Putative mammalian-associated viruses were identified by BLAST homology to known viral families and filtered for coverage > 75% and mapping identity > 90%, yielding 196 candidate vOTUs distributed across families including *Herpesviridae*, *Parvoviridae*, and *Poxviridae* (Supplementary Table S4). The majority of abundant viral sequences corresponded to dietary or environmental sources, including *Bee densovirus 2* in wild bats and *Zophobas morio black wasting virus* in captive bats, suggesting the presence of a closely related virus infecting an alternative worm host. High-coverage sequences included bat parvovirus 3 in *M. daubentonii* samples and a divergent papillomavirus in *N. noctula* (>1000× coverage), which may represent a novel bat-associated lineage pending further validation (Supplementary Table S5). No sequences related to known zoonotic viruses were detected.

## 4. Discussion

Our study provides an exploratory, cross-sectional baseline for the gut microbiome of insectivorous bats sampled in the wild and during temporary managed care. The microbiome appeared strongly individual-specific, and we detected no significant differences in overall prokaryotic community structure between wild and in-care bats, although host species, location, and care status are partially confounded and the analysis is therefore not a clean test of care effects. Among in-care bats, neither bacterial nor archaeal alpha diversity differed significantly with time in care, though the small group sizes limit the power of this comparison. These observations contrast with the reduced diversity and enrichment of opportunistic taxa commonly reported in captive mammals [5,25], and one possible contributing factor, to be tested in future work, is the use of whole-insect diets during rehabilitation, which more closely resemble natural prey than the processed or plant-based diets often provided to other captive mammals. Taken together, these cross-sectional patterns are consistent with a degree of microbiome stability during short-term managed care, but should be regarded as hypotheses for longitudinal testing rather than as evidence of resilience per se.

These observed patterns may be consistent with ecological and evolutionary traits associated with bats. Many species routinely experience fluctuating diets, variable foraging conditions, and rapid physiological shifts associated with heterothermy, which may select for flexible and disturbance-tolerant microbial communities [26,27]. The clear clustering of samples by host species rather than captivity status is consistent with patterns previously reported in studies describing phylosymbiosis in bats [8,26], although the current dataset was not designed to formally test phylogenetic effects. This pattern may reflect long-term interactions between host evolutionary history and microbiome composition, although dedicated phylogenetic analyses would be required to evaluate this hypothesis.

Microbiome stability in these bats may also reflect potential functional redundancy within gut microbial communities, a pattern observed across many vertebrates where core metabolic processes remain conserved even when taxonomic composition shifts [28]. Future functional metagenomic and metatranscriptomic analyses could determine whether nutrient-processing and immune-related functions remain conserved in rehabilitated bats. Together, these findings are consistent with the possibility of a resilient prokaryotic core, although functional profiling would be required to test this directly.

Dietary DNA analysis revealed expected differences between wild and captive bats, reflecting natural foraging versus standardised feeding in rehabilitation. Wild individuals consumed a diverse range of aquatic and woodland insects, whereas captive bats were fed primarily mealworms, with occasional supplementation. Correspondingly, several environmentally associated bacterial families were less abundant in bats that had spent longer in care, consistent with a simplified prey spectrum [28]. These changes occurred without detectable loss of overall bacterial diversity or alteration of archaeal community structure under the current sampling design. Dietary enrichment during rehabilitation may therefore contribute to the maintenance of microbial diversity and metabolic flexibility, although this will require direct testing.

Beyond bacteria and archaea, fungal and viral reads were detected but were largely environmental or dietary in origin. No sequences related to known zoonotic viruses were found. A divergent papillomavirus signal was identified in one *N. noctula* sample, but additional targeted sequencing would be needed to confirm whether it represents a true bat-associated virus.

From a conservation standpoint, the results suggest that temporary rehabilitation lasting up to 49 days was not associated with detectable large-scale disruption of microbiome composition under the current sampling conditions. If confirmed in future longitudinal studies, maintaining this microbial stability may be important because gut microbes contribute to nutrient assimilation, immune function, and post-release adaptation [6]. Microbiome profiling may therefore prove useful as an early indicator of rehabilitation success and welfare.

Several limitations of this study should be acknowledged. First, the cross-sectional sampling design means that each bat was sampled at a single time point, precluding longitudinal tracking of microbiome trajectories within individuals over the course of rehabilitation. Second, host species identity, roost location, feeding diet and rehabilitation status are partially confounded in this dataset, which limits the ability to disentangle the relative contributions of these factors to microbiome variation. In particular, rehabilitation status is confounded with host health, as bats admitted to care are injured or unwell whereas wild-roost individuals are presumed healthy; consequently, the absence of marked wild-versus-care differences cannot be attributed to managed care alone, and we did not test this contrast directly for the diversity and compositional analyses. Finally, as sampling was necessarily opportunistic within a rehabilitation context, group sizes were limited and uneven, which may constrain the detection of subtle microbiome differences and warrants cautious interpretation of small effect sizes. In addition, faecal pellets were collected non-invasively from surfaces beneath roosts or within enclosures, so the time between deposition and collection, and any associated environmental exposure or DNA degradation, could not be controlled and may have introduced variability. For these reasons, the present study is best regarded as exploratory, providing a cross-sectional baseline whose patterns require confirmation through longitudinal, controlled sampling rather than as a formal test of rehabilitation effects on the microbiome.

Future directions should include longitudinal tracking of individual bats from capture through release, integrating metagenomic, metabolomic, and physiological data. Linking microbial shifts to health markers, such as body condition, immune gene expression, and thermoregulatory capacity, will clarify functional consequences of captivity and reintroduction. Expanding comparative datasets across bat guilds (insectivorous, frugivorous, nectarivorous) will also test whether this apparent resilience is a broader chiropteran trait or specific to temperate insectivores.

## 5. Conclusions

Shotgun metagenomic sequencing of individual bat droppings provides a non-invasive framework for integrated assessment of host identity, diet, and microbial health. Despite simplified diets and captive conditions, insectivorous bats retained diverse microbial communities with no marked reductions in overall diversity or major shifts in community structure under the current sampling design. Longitudinal and functional studies will be required to determine whether these cross-sectional patterns reflect microbiome resilience. This contrasts with the pronounced microbiome alterations reported in many captive mammals and raises the possibility that bats may be relatively tolerant to short-term environmental and dietary variation. By documenting limited cross-sectional microbiome changes across bacterial and archaeal domains, our study highlights the potential utility of metagenomic profiling as a tool for monitoring bat welfare and rehabilitation outcomes. Future integration of functional metagenomics and longitudinal monitoring will strengthen the link between microbial resilience and ecological fitness, ensuring that rehabilitation efforts sustain not only the host but also its symbiotic microbiota.

## Data Availability

The original sequencing data presented in this study are openly available in the European Nucleotide Archive (ENA) under accession number PRJEB103765.

## Supplementary Materials

The following supporting information can be downloaded at: https://www.mdpi.com/article/10.3390/microorganisms14071403/s1, Supplementary Table S1. Overview of bat faecal samples by location and rehabilitation status; Supplementary Table S2. Host species identification from mitochondrial read mapping; Supplementary Table S3. Per-sample read counts through quality control and taxonomic classification; Supplementary Figure S1. Taxonomic classification efficiency of metagenomic reads in wild and rehabilitated bats; Supplementary Figure S2. Composition of the bat virome across all metagenomic samples; Supplementary Table S4. Viral families detected across all bat metagenomes; Supplementary Table S5. High-coverage viral sequences detected in individual samples.
