# Supplementary material for: Microbiome Stability in Wild and Rehabilitated Insectivorous Bats Revealed by Shotgun Metagenomics
Source: Microorganisms. 2026 Jun 25;14(7):1403. doi: 10.3390/microorganisms14071403 (PMC13413831; doi:10.3390/microorganisms14071403)
Supplement: Supplementary file 1 [file microorganisms-14-01403-s001.zip › microorganisms-4364537-supplementary.pdf]

Supplementary material

Supplementary Table S1. Overview of bat faecal samples by location and rehabilitation status

This table summarises faecal samples collected from wild bat roosts and rehabilitation centres in Yorkshire, UK. Samples are grouped by location/site, care status, and rehabilitation duration. For each site, the manuscript IDs of associated samples, total number of samples, and host species detected (based on mitochondrial confirmation; see Supplementary Table S2 and read mapping) are reported.

Location / Site	Area	Care status	Rehabilitation duration	Manuscript IDs	No. samples	Species detected
Dowley Gap	Bradford (West Yorkshire)	Wild roost	Baseline	W1, W2, W3, W4, W5	5	*Myotis daubentonii*
Dowley Gap 2	Bradford (West Yorkshire)	Wild roost	Baseline	W6, W7, W8, W9	4	*Myotis daubentonii*
Hirst Wood	Bradford (West Yorkshire)	Wild roost	Baseline	W10, W11, W12	3	*Pipistrellus pipistrellus*, *Nyctalus noctula*
Loft Roost	Keighley (West Yorkshire)	Wild roost	Baseline	W13, W14	2	*Pipistrellus pipistrellus*
DL Care facility	Kirklees (West Yorkshire)	Rehabilitation	≤7 days	C01, C02	2	*Myotis daubentonii*, *Nyctalus leisleri*
Fixby	Huddersfield area	Rehabilitation	≤7 days	C03	1	*Uncertain*
KW Care facility	North Yorkshire	Rehabilitation	1-49 days	C04, C05, C06, C07, C08, C09, C10, C11	8	*Pipistrellus pipistrellus*

Supplementary Table S2. Host species identification from mitochondrial read mapping

This table provides species-level host identification for all metagenomic faecal samples included in the study. For each sample, the top mitochondrial BLASTn hit (SSEQID), corresponding species, percent identity, and alignment length are shown. Species assignments were based on the highest-identity mitochondrial match (>95% identity and >500 bp alignment length).

Manuscript ID	Sample Name	Care Status	Rehabilitation duration	Location	SSEQID	Species Hit	Percent Identity	Alignment Length
**C01**	Care_DL_S17	Care	≤7 days	DL care facility				
**C02**	Care_DL_S18	Care	≤7 days	DL care facility	OZ183647.2	*Nyctalus leisleri*	99,81	15757
**C03**	Care_Fixby_S16	Care	≤7 days	Fixby				
**C04**	Care_KW_S20	Care	42 days	KW care facility	LR862378.1	*Pipistrellus pipistrellus*	99,929	15558
**C05**	Care_KW_S21	Care	45 days	KW care facility	LR862378.1	*Pipistrellus pipistrellus*	99,979	4807
**C06**	Care_KW_S22	Care	49 days	KW care facility	LR862378.1	*Pipistrellus pipistrellus*	99,929	15558
**C07**	Care_KW_S23	Care	4 days	KW care facility	LR862378.1	*Pipistrellus pipistrellus*	99,929	15559
**C08**	Care_KW_S24	Care	7 days	KW care facility	LR862378.1	*Pipistrellus pipistrellus*	99,929	15559
**C09**	Care_KW_S26	Care	1 day	KW care facility	LR862378.1	*Pipistrellus pipistrellus*	99,93	15640
**C10**	Care_KW_S28	Care	6 days	KW care facility	LR862378.1	*Pipistrellus pipistrellus*	99,93	15640
**C11**	Care_KW_S30	Care	1 day	KW care facility	LR862378.1	*Pipistrellus pipistrellus*	99,852	15580
**W1**	Wild_DG_S1	Wild	Baseline	Dowley Gap	MN122860.1	*Myotis daubentonii*	99.854	15757
**W2**	Wild_DG_S2	Wild	Baseline	Dowley Gap	MN122860.1	*Myotis daubentonii*	99.888	15134
**W3**	Wild_DG_S3	Wild	Baseline	Dowley Gap	OY725382.2	*Myotis daubentonii*	99.749	15919
**W4**	Wild_DG_S5	Wild	Baseline	Dowley Gap	MN122860.1	*Myotis daubentonii*	99.754	15838
**W5**	Wild_DG_S6	Wild	Baseline	Dowley Gap				
**W6**	Wild_DG2_S10	Wild	Baseline	Dowley Gap 2				
**W7**	Wild_DG2_S12	Wild	Baseline	Dowley Gap 2				
**W8**	Wild_DG2_S7	Wild	Baseline	Dowley Gap 2	MN122860.1	*Myotis daubentonii*	99.874	15822
**W9**	Wild_DG2_S8	Wild	Baseline	Dowley Gap 2	MN122860.1	*Myotis daubentonii*	99.911	15702
**W10**	Wild_HirstWood_S13	Wild	Baseline	Hirst Wood				
**W11**	Wild_HirstWood_S14	Wild	Baseline	Hirst Wood	MN122907.1	*Nyctalus noctula*	99.815	15648
**W12**	Wild_HirstWood_S15	Wild	Baseline	Hirst Wood	MN122907.1	*Nyctalus noctula*	99.802	15648
**W13**	Wild_LoftRoost_S31	Wild	Baseline	Loft Roost	LR862378.1	*Pipistrellus pipistrellus*	99.955	15558
**W14**	Wild_LoftRoost_S32	Wild	Baseline	Loft Roost	LR862378.1	*Pipistrellus pipistrellus*	99.904	15558

Supplementary Table S3. Per-sample read counts through quality control and taxonomic classification.

Raw reads, reads after human host removal (Hostile v2.0.0), and post-QC reads (after adapter/quality trimming with TrimGalore v0.6.10 and host removal) are reported for the 32 bat faecal extracts initially processed, together with one mealworm (Tenebrio molitor) reference sample. The percentage of reads classified and unclassified by Kraken2 (k2_core_nt_20250609) is shown for retained samples. Seven bat extracts yielding fewer than 1 million post-QC reads (Care_KW_S25, Care_KW_S27, Wild_DG_S4, Wild_DG2_S11, Wild_LoftRoost_S29, Wild_LoftRoost_S33, Wild_DG_S9) were excluded from downstream analysis and are not assigned a manuscript ID; the remaining 25 samples (14 wild, 11 in care) were retained.

		QUALITY CONTROL			KRAKEN2 PROFILING	
**MANUSCRIPT ID**	Sample_short	raw_reads	human_filtered	Post_QC	unclassified_percent	classified_percent
**C01**	Care_DL_S17	363804932	363197724	362273906	2.010464425	97.98953558
**C02**	Care_DL_S18	572260370	572116400	571267254	12.44745213	87.55254787
**C03**	Care_Fixby_S16	259390586	259176766	258816570	34.37982815	65.62017185
**C04**	Care_KW_S20	334791542	334784080	333620824	1.338924815	98.66107519
**C05**	Care_KW_S21	14210402	13864186	13837992	28.59601306	71.40398694
**C06**	Care_KW_S22	143083472	143051914	142874902	62.36341775	37.63658225
**C07**	Care_KW_S23	1583149034	1583064432	1577814450	2.141292343	97.85870766
**C08**	Care_KW_S24	347875380	347668234	346836442	11.23150087	88.76849913
	Care_KW_S25	0	0	0		
**C09**	Care_KW_S26	48797018	48771884	48674442	11.92841615	88.07158385
	Care_KW_S27	0	0	0		
**C10**	Care_KW_S28	252413884	252346774	251788698	3.871023631	96.12897637
**C11**	Care_KW_S30	294796410	294018464	293550064	45.92441853	54.07558147
**W1**	Wild_DG_S1	67736080	67614436	67508660	69.10814701	30.89185299
**W2**	Wild_DG_S2	82664368	82434840	82311580	67.6830599	32.3169401
**W3**	Wild_DG_S3	94262360	94188690	94043608	64.6713916	35.3286084
	Wild_DG_S4	0	0	0		
**W4**	Wild_DG_S5	150027032	149873770	149623436	47.04139664	52.95860336
**W5**	Wild_DG_S6	42522838	42365040	42304316	59.11479103	40.88520897
	Wild_DG_S9	30760	30758	30736	55.73269131	44.26730869
**W6**	Wild_DG2_S10	10345042	10344536	10332316	23.27809177	76.72190823

	Wild_DG2_S11	0	0	0		
**W7**	Wild_DG2_S12	251027054	250996534	250322780	54.6978569	45.3021431
**W8**	Wild_DG2_S7	376968856	376591288	375925324	51.82415591	48.17584409
**W9**	Wild_DG2_S8	401429620	398463042	397924520	49.97148102	50.02851898
**W10**	Wild_HurstWood_S13	101056082	101053938	100886274	30.03818141	69.96181859
**W11**	Wild_HurstWood_S14	251884492	243337750	242984134	18.99453402	81.00546598
**W12**	Wild_HurstWood_S15	423580302	422260052	421385270	46.56732543	53.43267457
	Wild_LoftRoost_S29	2	2	2		
**W13**	Wild_LoftRoost_S31	416619154	416469512	415951166	53.8489487	46.1510513
**W14**	Wild_LoftRoost_S32	484107960	483952346	483338656	56.85284522	43.14715478
	Wild_LoftRoost_S33	0	0	0		

Supplementary Figure S1. Taxonomic classification efficiency of metagenomic reads in wild and rehabilitated bats

A) Total number of classified and unclassified reads per sample following Kraken2 taxonomic assignment. Bars represent individual metagenomes (n = 26), with classified reads shown in turquoise and unclassified reads in red. Samples include both wild bats and individuals in rehabilitation.
B) Proportion of unclassified reads (%) in rehabilitated (Care) and wild bats. Wild samples showed a significantly higher proportion of unclassified reads than rehabilitated bats (Wilcoxon rank-sum test, p = 0.014), consistent with increased dietary and environmental diversity in wild conditions.
C) Alpha-diversity (Shannon index at the species level) in the bats in rehabilitation grouped by their stay length in care

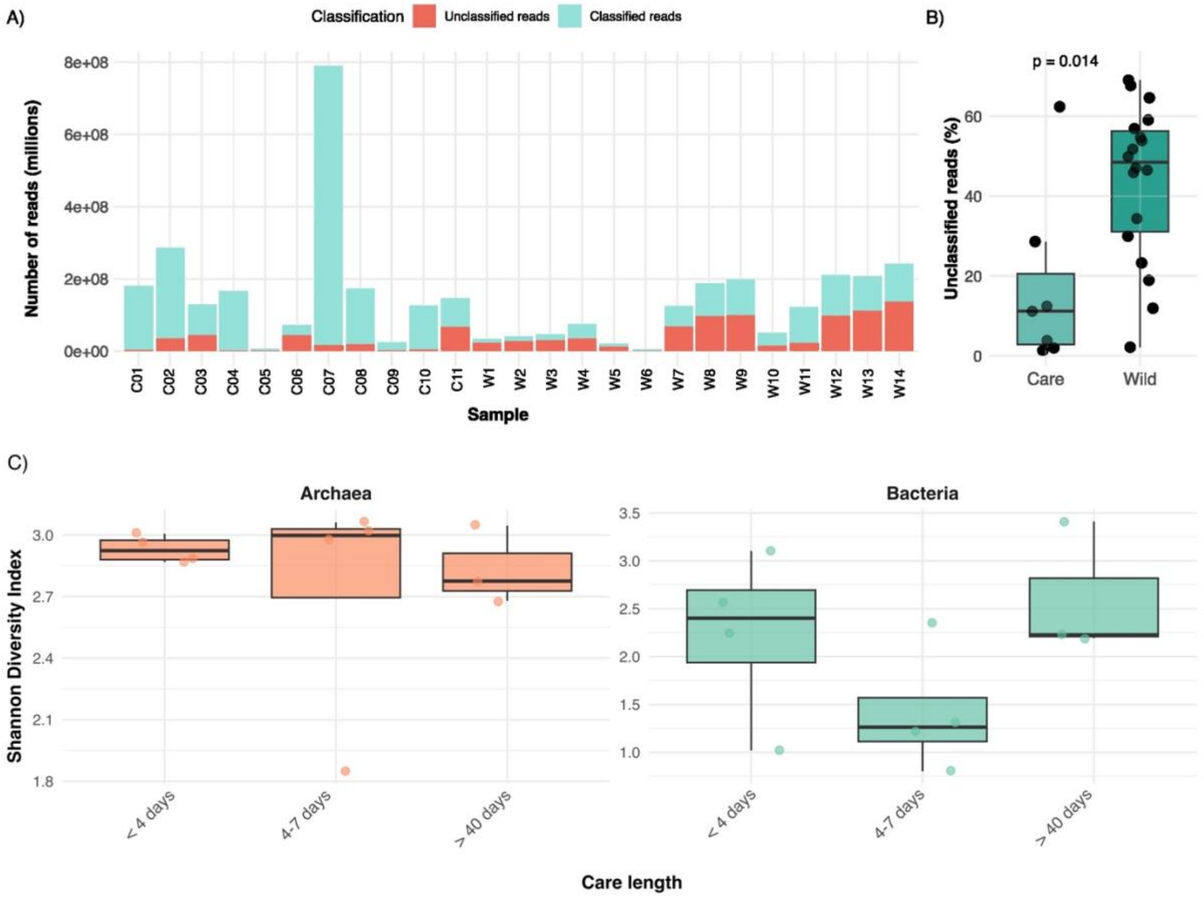

Supplementary Figure S2. Composition of the bat virome across all metagenomic samples. Distribution of viral operational taxonomic units (vOTUs) detected across all 26 bat metagenomes grouped by viral realm. The virome was dominated by bacteriophages of the realm Duplodnaviria (20,565 vOTUs; 90.5%), followed by smaller contributions from Varidnaviria, Monodnaviria, and Riboviria. A minority of vOTUs were unclassified or belonged to unranked viral groups. The predominance of phage-associated realms reflects the bacterial origin of most viral sequences within gut metagenomes.

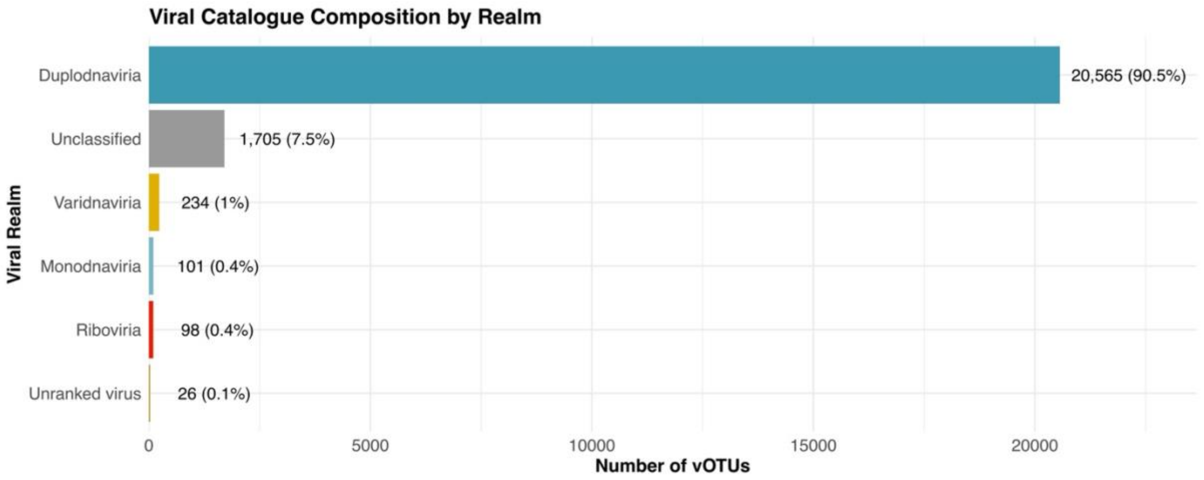

Supplementary Table S4. Viral families detected across all bat metagenomes

Summary of viral operational taxonomic units (vOTUs) assigned to each viral family across the 26 metagenomic samples. Viral families are grouped by ecological or host association: mammalian viruses, invertebrate viruses, fungal viruses, environmental viruses, and arthropod/plant viruses. For each family, vOTUs and their percentage contribution to the total viral catalogue are reported. These values reflect the expected predominance of dietary, environmental, and bacteriophage-derived sequences in gut metagenomes.

Viral Family	Number of vOTUs	% of Catalogue
**Mammalian virus**	196	0,87
**Herpesviridae**	43	0,19
**Retroviridae**	41	0,18
**Poxviridae**	39	0,17
**Parvoviridae**	36	0,16
**Papillomaviridae**	16	0,07
**Adenoviridae**	13	0,06
**Polyomaviridae**	8	0,04
**Invertebrate virus**	92	0,4
**Adintoviridae**	39	0,17
**Iridoviridae**	30	0,13
**Baculoviridae**	23	0,1
**Fungal virus**	25	0,11
**Totiviridae**	18	0,08
**Partitiviridae**	7	0,03
**Environmental virus**	88	0,38
**Mimiviridae**	51	0,22
**Phycodnaviridae**	30	0,13
**Lavidaviridae**	7	0,03
**Arthropod/Plant virus**	8	0,04
**Phenuiviridae**	8	0,04

Supplementary Table S5. High-coverage viral sequences detected in individual samples. Viral operational taxonomic units (vOTUs) achieving notable sequencing coverage (>50×) in individual bat samples. Columns include sample identifier, confirmed bat species, inferred viral realm, sequencing coverage, and closest reference match. High-coverage viral hits included Parvoviridae-like sequences
(Parvovirus bat6352) in several wild *Myotis daubentonii* and *Nyctalus noctula* samples, and two Duplodnaviria hits corresponding to endogenous bat chromosomal sequences in *Pipistrellus pipistrellus*.

ID	Sample_short	Bat	Realm	Coverage	Closest_hit
C10	Care_KW_S28	Pipistrellus pipistrellus	Duplodnaviria	100	bat chromosome
C04	Care_KW_S20	Pipistrellus pipistrellus	Duplodnaviria	82	bat chromosome
C05	Care_KW_S21	Pipistrellus pipistrellus	Monodnaviria	4108	
W12	Wild_Hirst Wood_S15	Nyctalus noctula	Monodnaviria	1405	Parvovirus bat6352
W2	Wild_DG_S2	Myotis daubentonii	Monodnaviria	94	Parvovirus bat6352
W3	Wild_DG_S3	Myotis daubentonii	Monodnaviria	53	Parvovirus bat6352
W12	Wild_Hirst Wood_S15	Nyctalus noctula	Monodnaviria	12436	
W3	Wild_DG_S3	Myotis daubentonii	Monodnaviria	347	Parvovirus bat6352
W12	Wild_Hirst Wood_S15	Nyctalus noctula	Monodnaviria	285	Parvovirus bat6352
W2	Wild_DG_S2	Myotis daubentonii	Monodnaviria	58	Parvovirus bat6352
W2	Wild_DG_S2	Myotis daubentonii	Monodnaviria	92	
C05	Care_KW_S21	Pipistrellus pipistrellus	Monodnaviria	300	Protoparvovirus chiropteran2
C04	Care_KW_S20	Pipistrellus pipistrellus	Monodnaviria	54	Protoparvovirus chiropteran2
C01	Care_DL_S17	Nyctalus leisleri	Monodnaviria	8306177	Zophobas morio black wasting virus
C04	Care_KW_S20	Pipistrellus pipistrellus	Monodnaviria	7439284	Zophobas morio black wasting virus
C10	Care_KW_S28	Pipistrellus pipistrellus	Monodnaviria	5023581	Zophobas morio black wasting virus
C05	Care_KW_S21	Pipistrellus pipistrellus	Monodnaviria	21425	Zophobas morio black wasting virus
W12	Wild_Hirst Wood_S15	Nyctalus noctula	Monodnaviria	192	Zophobas morio black wasting virus
W3	Wild_DG_S3	Myotis daubentonii	Monodnaviria	9271	Protoambidensovirus incertum4
W3	Wild_DG_S2	Myotis daubentonii	Monodnaviria	121	Protoambidensovirus incertum4
W3	Wild_DG_S3	Myotis daubentonii	Monodnaviria	57	Bee densovirus 2
W3	Wild_DG_S3	Myotis daubentonii	Monodnaviria	2690	Bee densovirus 2
W2	Wild_DG_S2	Myotis daubentonii	Monodnaviria	238	Bat parvovirus 3
W2	Wild_DG_S2	Myotis daubentonii	Riboviria	58	NA
W2	Wild_DG_S2	Myotis daubentonii	Riboviria	91	bat chromosome
